# A rare case report of primary ovarian carcinoid presenting with constipation

**DOI:** 10.3389/fonc.2025.1489978

**Published:** 2025-02-06

**Authors:** Xiaofeng Deng, Qian Huang, Bangfang Xie, Hailong Huang, Jianguo Chen

**Affiliations:** ^1^ Department of Gynaecology and Obstetrics, Chengdu Qingbaijiang District People’s Hospital, Chengdu, Sichuan, China; ^2^ Department of Pathology, Chengdu Qingbaijiang District People’s Hospital, Chengdu, Sichuan, China; ^3^ Department of Medical Laboratory, Chengdu Qingbaijiang District People’s Hospital, Chengdu, Sichuan, China

**Keywords:** carcinoid, primary ovarian carcinoid, ovary, case report, constipation

## Abstract

**Background:**

Ovarian carcinoids are exceptionally rare. This report discusses an unprecedented case of a primary ovarian mixed insular and trabecular carcinoid, presenting with constipation.

**Case Presentation:**

A 47-year-old female presented with a four-month history of constipation. A comprehensive abdominal CT with contrast revealed a large mass in the pelvic region, measuring 8.6×9.7×9.3cm. Consequently, the patient was referred for further evaluation. Intraoperative exploration uncovered a 10.0×9.0 cm mass in the left ovary. Initial histopathological assessment suggested a sex cord-stromal cell tumor, leading to a left ovariectomy and bilateral salpingectomy. Final histopathological analysis post-surgery identified the mass as a mixed insular and trabecular carcinoid. The patient was diagnosed with stage Ia, T1aN0M0 primary ovarian mixed insular and trabecular carcinoid.

**Conclusions:**

Diagnosis of carcinoids predominantly relies on postoperative histopathological examination. As of now, There is no established standard treatment, emphasizing the necessity for ongoing patient monitoring.

## Introduction

Carcinoids, rare and typically slow-growing neuroendocrine tumors, often remain clinically silent until metastasis or the emergence of carcinoid syndrome. Frequently discovered incidentally, these neoplasms are most commonly found in the gastrointestinal tract and lungs (1), with occasional occurrences in the kidneys. Carcinoids originating from reproductive organs, particularly the ovaries, are exceedingly rare, constituting only 1% of all carcinoid cases (2). Ovarian carcinoids are typically classified into four types: insular, trabecular, strumal, and mucinous, with each exhibiting distinct histological characteristics. Mixed carcinoids, comprising two or more histological patterns, have been sporadically reported in recent years, demonstrating varied clinical behaviors and prognoses (3). Due to the limited number of reported cases, the clinical behaviors of mixed carcinoid subtypes remain underexplored. To the best of our knowledge, persistent constipation has not been reported to be the main clinical manifestation in primary ovarian mixed insular and trabecular carcinoid.

## Case report

**Figure 1:**
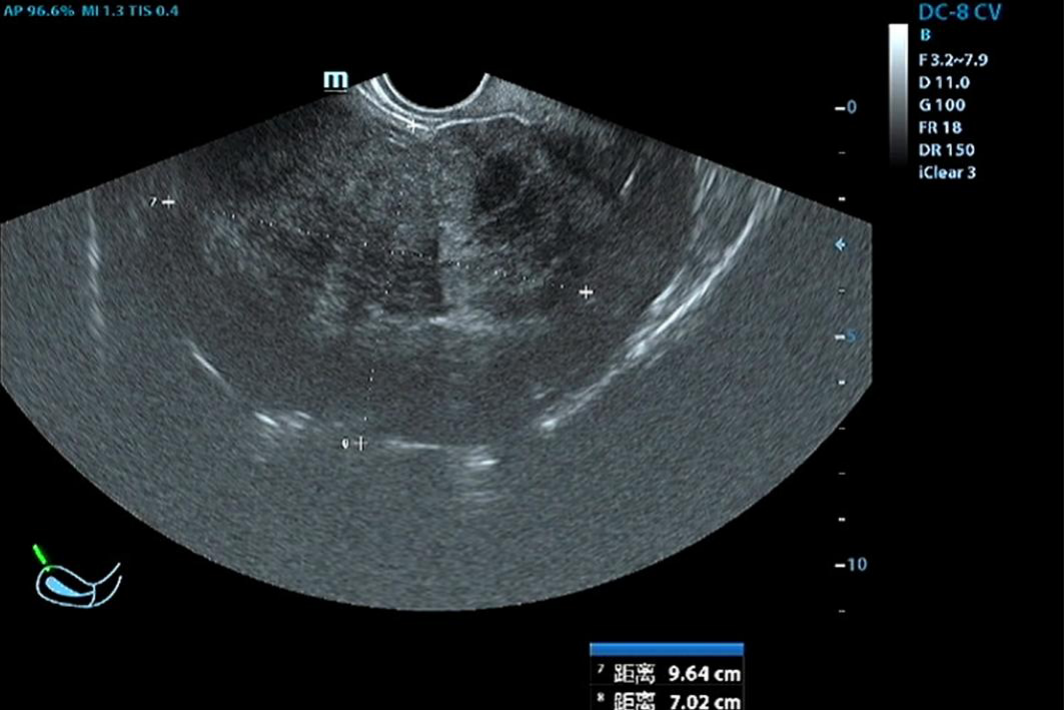
Vaginal Doppler Ultrasonography: Reveals a large pelvic mass measuring 9.6 × 7.0 × 10.8 cm, indicative of a significant space-occupying lesion.

**Figure 2:**
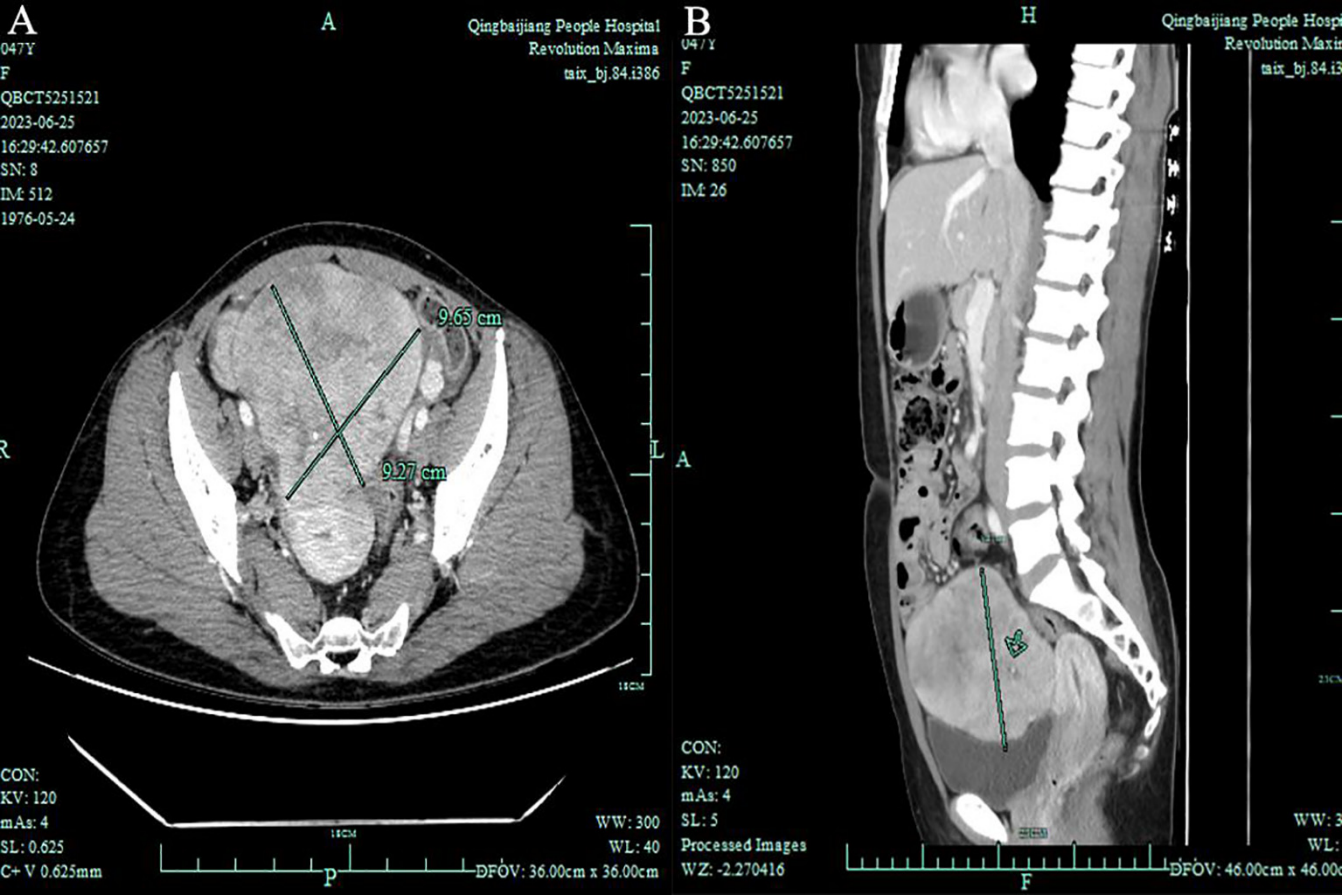
Total Abdominal Contrast CT Imaging: **(A)** Axial view - Demonstrates a pelvic space-occupying lesion measuring 8.6 × 9.7 × 9.3 cm, with indistinct posterior margins relative to the uterus. **(B)** Sagittal view - Highlights the lesion’s extent within the pelvic cavity without evident metastasis to the omentum or para-aortic lymph nodes.

In June 2023, a 47-year-old woman sought medical advice for persistent constipation lasting four months. An abdominal CT scan revealed a large pelvic tumor, approximately 9.5 cm in diameter, raising suspicion for a neoplastic growth. The patient, with a gravidity of 4 and parity of 3, had no significant family history of chronic diseases. Physical and gynecological examinations were unremarkable, failing to detect the tumor. Laboratory tests, including serum tumor markers (CEA, CA125, CA153) and calcium levels, were within normal ranges. Vaginal ultrasound presented a homogeneous uterine wall, and color Doppler flow imaging (CDFI) showed no abnormal ovarian blood flow. However, a solid pelvic mass with regular morphology and clear margins was noted, initially suspected as a subserous myoma by radiologists(**Figure 1**). Subsequent abdominal CT highlighted a space-occupying lesion in the pelvic cavity, poorly demarcated from the uterus, with no evidence of metastasis (**Figure 2**).

**Figure 3:**
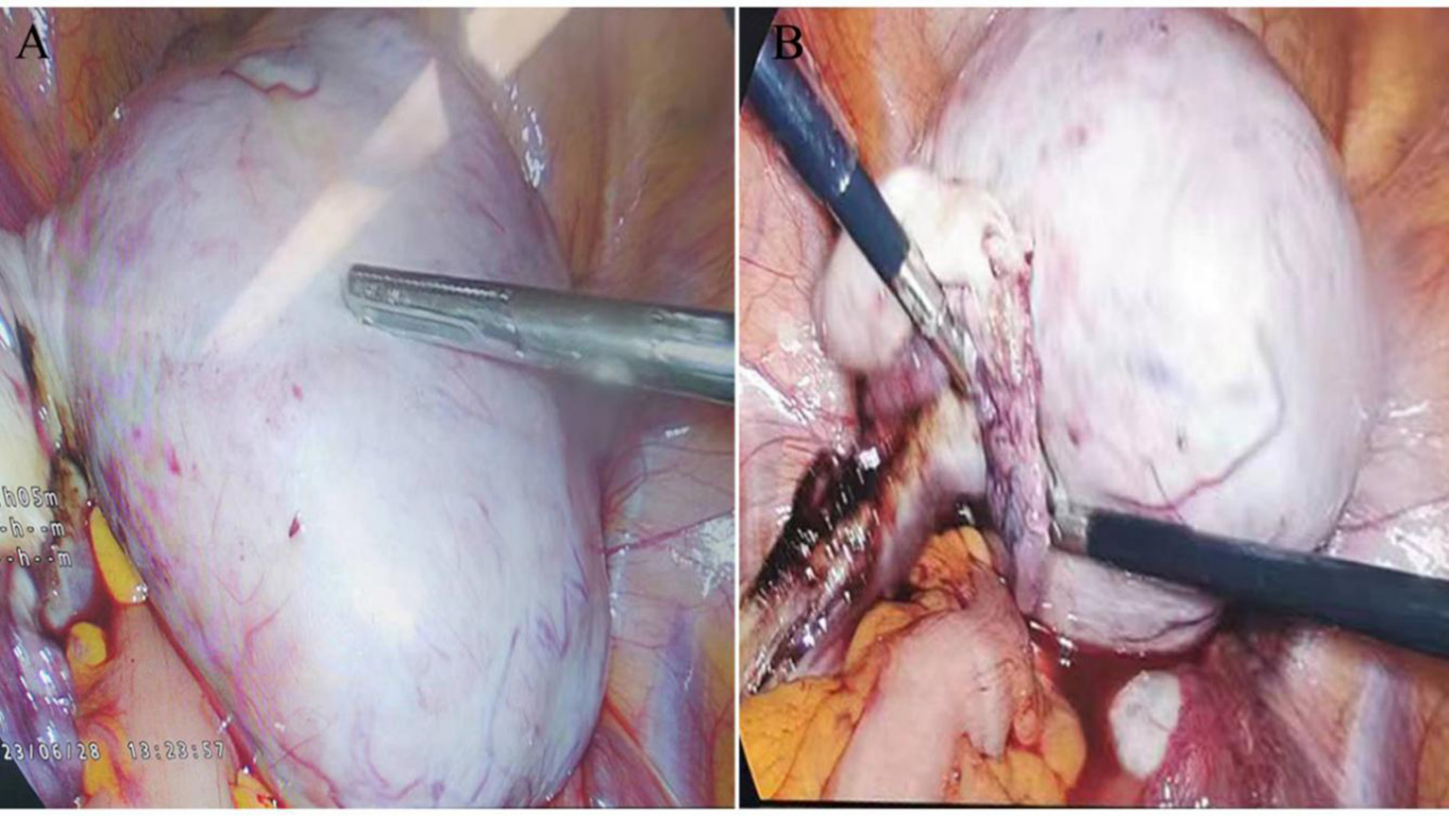
Intraoperative Laparoscopic Images: **(A)** Shows a 10.0 × 9.0 cm tumor in the left ovary, characterized by an endophytic growth, an intact capsule, and clear boundaries. **(B)** Illustrates the absence of adhesions to surrounding tissues and the lack of visible solid tumor signs on the omentum or peritoneum.

**Figure 4:**
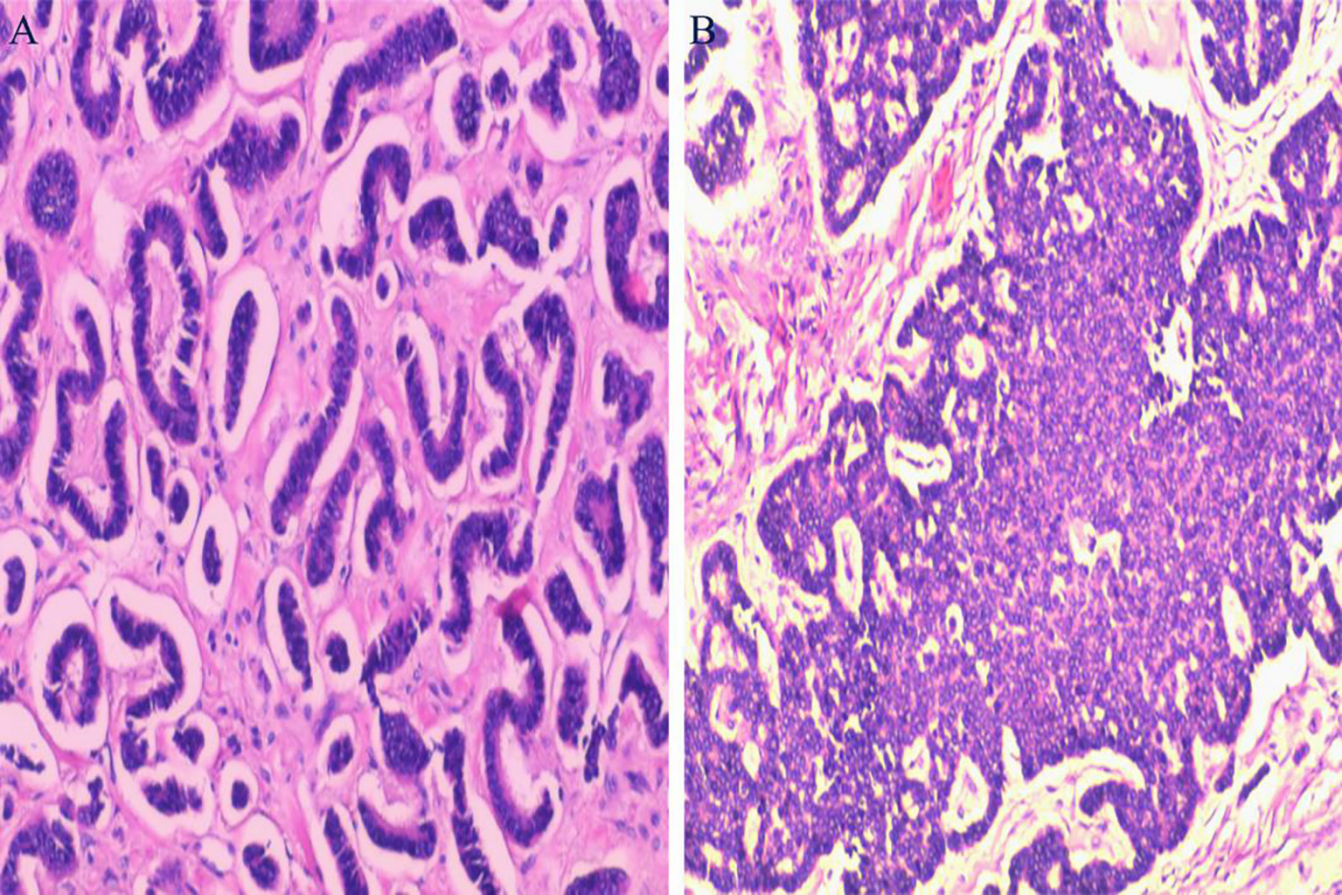
Histopathological Examination: **(A)** Displays tumor cells arranged in insular patterns. **(B)** Shows tumor cells arranged in trabecular patterns. (Hematoxylin and Eosin staining, magnification ×100).

**Figure 5:**
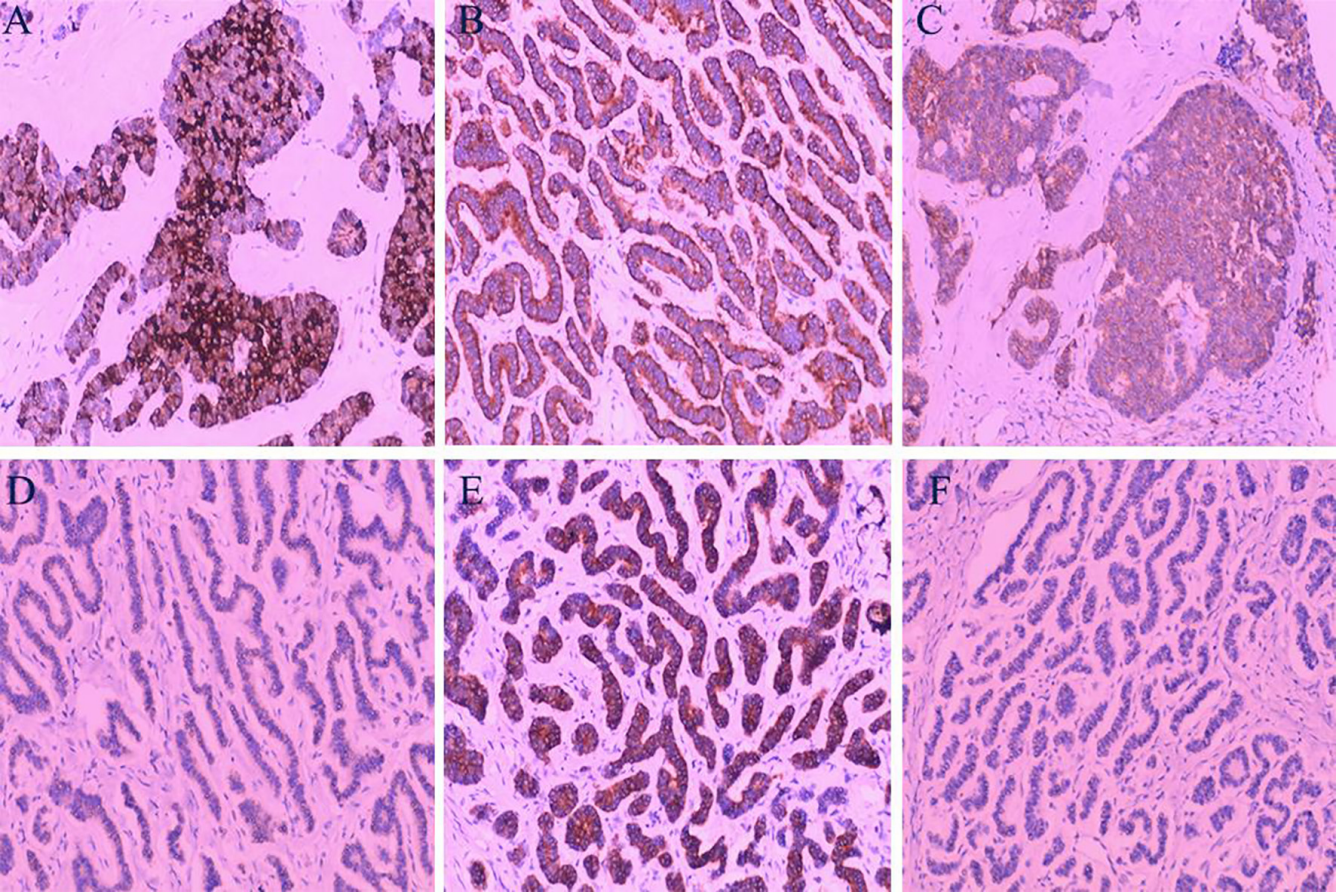
Immunohistochemical Analysis: **(A)** Tumor cells demonstrating strong positivity for Chromogranin A (CgA). **(B)** Tumor cells showing strong positivity for Synaptophysin (Syn). **(C)** Positive staining for CD56 in tumor cells. **(D)** Negative staining for GATA3 in tumor cells. **(E)** Strong positivity for AE1/AE3 in tumor cells. **(F)** Tumor cells negative for PAX-8. (Hematoxylin and Eosin staining, magnification ×100).

During exploratory laparotomy, a 10.0×9.0 cm tumor was discovered in the left ovary, with an intact capsule and clear boundaries, devoid of adhesions or peritoneal involvement. The uterus appeared normal, with no surface nodules (**Figure 3**). The intraoperative frozen section histopathological examination suggested a sex cord-stromal cell tumor, leading to a left ovariectomy and bilateral salpingectomy while preserving the uterus. Postoperatively, the patient recovered well, with prompt relief from constipation and discharge on the 7th day without complications. Detailed histopathological examination revealed a mixed insular and trabecular carcinoid. (**Figure 4**). The results of immunohistochemical staining showed that the structures were positive for chromogranin A (CgA), synaptophysin, AE1/AE3, CD56, and vimentin, and negative for calretinin, α-inhibin, GATA-3, TTF-1, PAX-8, EMA, CK7, P53, CD10, WT-1, ER, and PR. Besides, the positive index of Ki67 was 5% (**Figure 5**). Confirmed through immunohistochemical staining, the patient was diagnosed with a primary ovarian carcinoid, FIGO stage Ia, T1aN0M0. No chemoradiotherapy or secondary surgery was undertaken, and a follow-up abdominal CT at five months post-surgery showed no signs of recurrence or metastasis.

## Discussion

Primary ovarian carcinoids, although rare and accounting for less than 0.1% of all ovarian tumors, can affect women across a wide age range, often presenting unilaterally. Common symptoms include an enlarging abdominal mass, distension, or abnormal uterine bleeding (4). The diagnosis of ovarian carcinoids is challenging due to their non-specific clinical manifestations and lack of distinctive biological markers. Radiologically, these solid tumors are indistinguishable from primary solid or metastatic ovarian cancers, necessitating reliance on postoperative pathological examination for definitive diagnosis.

Approximately 10% of patients with ovarian carcinoids exhibit carcinoid syndrome, characterized by symptoms such as flushing, diarrhea, asthma, and heart damage, which can aid in earlier diagnosis (5). The four major histological types of primary ovarian carcinoids—insular, trabecular, strumal, and mucinous—display varied clinical manifestations and prognoses. Malignancy is more commonly associated with mucinous or insular types. Notably, the incidence of carcinoid syndrome differs significantly between trabecular and insular types.

The patient in this case exhibited constipation as the sole clinical symptom, with normal tumor markers (CEA, CA125, CA153). This presentation differs from typical strumal ovarian carcinoids, which often manifest with long-term constipation. The involvement of peptide YY, a substance derived from carcinoid tissues known to inhibit gastrointestinal motility, might explain the constipation observed in such cases (6–8).

Currently, there are no standardized treatment guidelines for ovarian carcinoids. Early-stage carcinoids, especially of the insular or trabecular type, are typically managed with total abdominal hysterectomy and bilateral salpingo-oophorectomy. Mucinous types may require additional omentectomy and para-aortic lymphadenectomy. Evidence suggests that uterus preservation and unilateral salpingo-oophorectomy do not adversely affect survival in stage I disease. Treatment for recurrent and metastatic carcinoids includes secondary surgical resection, adjuvant chemotherapy, radiotherapy, and molecular therapies (9).

The prognosis of primary ovarian carcinoid is contingent upon a number of factors, including pathological stage, histologic subtype, and proliferative activity. The presence of carcinoid syndrome is also a significant indicator of a poorer outcome. The prognosis is extraordinarily good in the early stage, the 10-year survival rate in stage I primary ovarian carcinoid patients is as high as 100%, whereas the 5-year survival rate in the later stage decreases to 33% (10).The insular, trabecular, and stromal carcinoids of primary ovarian carcinoid exhibit better prognosis than mucinous types (11). The presence of carcinoid syndrome indicates a poorer outcome. Ki-67, a marker of tumor aggressiveness, is used for grading neuroendocrine tumors and predicting prognosis. A higher Ki-67 index correlates with a worse prognosis (12). In this case, with a Ki-67 index of 5% and no metastasis observed on contrast CT, the patient may have a favorable survival outlook. However, close follow-up is essential due to the variability in clinical outcomes among different carcinoid types and the rarity of cumulative experience with this disease.

## Conclusions

The challenges in preoperative diagnosis of ovarian carcinoids stem from their non-specific symptoms and radiological similarities to other ovarian neoplasms. Currently, there is no established standard treatment protocol for ovarian carcinoids, highlighting the necessity for individualized patient management. Regular follow-up is crucial for monitoring disease progression and ensuring effective control.

## Data Availability

The raw data supporting the conclusions of this article will be made available by the authors, without undue reservation.
